# Agreement between QIAreach and QuantiFERON-TB Gold Plus for detecting TB infection in household contacts

**DOI:** 10.5588/ijtldopen.26.0431

**Published:** 2026-11-13

**Authors:** G.K. Daffeh, I.L. Manneh, A.F. Cox, A. Barry, A. Gindeh, M. Rashid, O. Cham, O. Owolabi, J.S. Sutherland

**Affiliations:** Vaccines and Immunity Theme, MRC Unit The Gambia at LSHTM, Fajara, The Gambia.

**Keywords:** tuberculosis, The Gambia, IGRA, TBI, time-to-result

Dear Editor,

Identifying TB infection (TBI) for targeted preventative therapy is a cornerstone in the broad strategy to end TB,^1^ yet point-of-care diagnostics for TBI remain scarce in high-burden settings where they are most needed. In West Africa, the prevalence of TBI is believed to be between 19.5% and 22.8% across different populations, and The Gambia’s rate is estimated around 18.7%.^2^ While interferon-gamma release assays (IGRAs) are widely used for TBI testing in high-income countries, their laboratory requirements and complexity can limit deployment in lower-resource settings, where the tuberculin skin test remains widely used despite its limited specificity for Mycobacterium tuberculosis infection and susceptibility to false-positive results. The QIAreach QuantiFERON-TB test (QIAGEN, Germany), a portable and rapid digital IGRA platform, may help address these barriers; however, evidence of its performance in TB-endemic African settings remains limited.

We performed a head-to-head comparison of the QIAreach QuantiFERON-TB and QuantiFERON-TB Gold Plus (QFT-Plus) among household contacts of bacteriologically confirmed pulmonary TB cases in The Gambia. This study was nested within the TrENDx-TB study.^3^ Ethical statement: the study was approved by the MRC/Gambian government joint ethics committee (LEO21727). We further assessed immune responses associated with TBI by profiling cytokines in QIAreach-stimulated supernatants to identify potential TB biomarkers.

Forty-four HIV-negative adult and adolescent household contacts (median [IQR] age, 19.5 [16–30.25] years) of individuals with bacteriologically confirmed pulmonary TB were enrolled following written informed consent and/or assent. Household contacts were individuals who lived in the same house or compound as an index TB case for at least three months before recruitment. Blood collected in lithium-heparin tubes was processed in parallel using the QIAreach QuantiFERON-TB and QuantiFERON-TB Gold Plus (QFT-Plus) platforms. For QIAreach testing, 1 mL of whole blood was incubated overnight in a dedicated stimulation tube and subsequently analysed using the QIAreach eHub platform (QIAGEN, Germany), which generated qualitative results for up to eight samples within 20 min after incubation. QFT-Plus testing was performed according to the manufacturer’s instructions, with interferon-gamma (IFN-γ) quantified by ELISA following overnight stimulation.

We observed substantial agreement between the two tests (Table), with positive percent agreement of 91.7% (11/12), negative percent agreement of 87.5% (28/32), and overall percent agreement of 88.6% (39/44; Cohen’s κ = 0.73). Previous studies from low-incidence settings, including Japan, Malaysia, and the USA, reported high concordance between QIAreach and QFT-Plus, generally exceeding 90%.^4–6^ However, evidence from high-TB-burden African settings remains limited and has been more heterogeneous. Ntshiqa et al.^7^ reported moderate agreement between QIAreach and QFT-Plus (78.4%; κ = 0.56) among predominantly adult household contacts across three high-TB-burden African countries, while a community-based study in Malawi (n = 1,049), which predominantly enrolled children, reported lower agreement (κ = 0.26), with sensitivity and specificity of 62% and 74%, respectively.^8^ A study from Zambia further highlighted portability and operational simplicity of QIAreach, supporting its potential utility for TBI testing in resource-limited settings.^9^ Our findings of substantial agreement in a predominantly adolescent and adult household-contact population add to emerging evidence supporting the use of QIAreach in high-burden settings. The heterogeneity in agreement across studies may reflect differences in age distribution, HIV status, underlying TB epidemiology, and study design, and warrants further evaluation in larger, diverse African populations.

**Table.** Comparing test performance.

Test performance	Frequency	% Agreement
Positive percent agreement	11/12	91.67%
Negative percent agreement	28/32	87.50%
Overall percent agreement	39/44	88.60%
Kappa agreement	0.73	

Five participants had discordant results between the two assays. Four of these were QIAreach-positive/QFT-Plus-negative and one was QIAreach-negative/QFT-Plus-positive. All four QIAreach-positive/QFT-Plus-negative results occurred among participants aged 14–25 years, with an equal sex distribution (two male and two female). Three of these participants had low or negative quantitative IFN-γ responses for both QFT-Plus (0.08, −0.02, 0.05 IU/mL) and QIAreach (−0.04, 0.04, 0.09 IU/mL). The fourth had a quantitative IFN-γ response of 0.77 IU/mL for QIAreach but no detectable QFT-Plus response (0 IU/mL). Conversely, the QIAreach-negative/QFT-Plus-positive participant was a 46-year-old female. Quantitative IFN-γ response was 5.33 IU/mL for QFT-Plus and 0.12 IU/mL for QIAreach. None of the participants with discordant results had clinical features suggestive of TB that could readily explain the discrepant findings. Thus, reason for discordance remains uncertain, particularly in the absence of an independent reference standard for confirming TBI.

Interestingly, the time-to-result (TTR) of QIAreach varied across the discordant samples, ranging from 10.08 to 20 min. The QIAreach TTR inversely correlated with IFN-γ magnitude across the study population (Spearman’s ρ = −0.72, *P* < 0.0001). Stronger IFN-γ responses associated with quicker results, whereas weak responses or negative responses generally had longer TTRs. Although TTR is not an established TB diagnostic measure, its association with IFN-γ magnitude suggests potential indicator of response strength, but its clinical relevance requires further investigation.

Additional immune signatures associated with QIAreach positivity were explored by multiplex cytokine profiling using an existing Bio-Rad 48-plex human cytokine assay on 22 participants, pragmatically selected based on availability of stimulated supernatants. Seven analytes were significantly associated with QIAreach-positive responses: IL-2, IP-10 (CXCL10), IFN-γ, MIG (CXCL9), MIP-1β (CCL4), IL-1RA, and IL-13. Among these, IL-2 (*P* < 0.0001, median [IQR]: 242.7 [130.5–366.5], area under the curve [AUC] = 1.000) and MIG (*P* < 0.0001, median [IQR]: 19,431 [6,666–27,354], AUC = 1.000) strongly associated with QIAreach positivity and are of particular interest given their roles in T-cell activation and IFN-γ-associated immune signalling, respectively, supporting further investigation of multi-biomarker approaches for TB immunodiagnostics.^10,11^ However, these exploratory findings require validation across larger, diverse populations to establish biomarker utility and potential integration into immunodiagnostic platforms.

We acknowledge important limitations. The sample size (n = 44) was small to draw definitive conclusions about diagnostic accuracy, particularly for borderline and discordant results. The absence of longitudinal follow-up prevented assessment of subsequent progression to active TB disease. Our study also lacked non-TB exposed controls and immunocompromised participants, including people living with HIV and individuals receiving immunosuppressive therapies, limiting the generalisability of our findings in these populations.^12^ Finally, the cytokine analysis was restricted to a small subset of participants with available supernatants, limiting the power to identify and validate potential biomarkers.

In our TB-endemic West African cohort, QIAreach showed 88.6% concordance with QFT-Plus, providing preliminary evidence of substantial agreement between the two platforms. Its portability, simplified workflow, and rapid readout may offer operational advantages for decentralised TBI testing in settings where conventional laboratory-based IGRAs are difficult to implement. However, larger, multi-site studies across diverse African populations, including younger children and people living with HIV, are needed to evaluate performance across different epidemiological and clinical contexts. Nonetheless, field evaluations provide valuable evidence supporting simplified IGRA implementation in TB-endemic settings, where conventional laboratory access is limited and expanded testing among all at-risk populations remains essential.^13,14^
